# Analysis of 12 cases of antineoplastic agents-induced interstitial lung disease

**DOI:** 10.3389/fphar.2023.1218480

**Published:** 2023-08-28

**Authors:** Xiao Li, Yong-Li Gu, Xu-Chao Liu, Zeng-Xian Sun, Ying Sun

**Affiliations:** ^1^ Department of Pharmacy, The First People’s Hospital of Lianyungang, Lianyungang, China; ^2^ Jiangsu Key Laboratory of New Drug Research and Clinical Pharmacy, Xuzhou Medical University, Xuzhou, China

**Keywords:** antineoplastic agents, drug-induced interstitial lung diseases, immune checkpoint inhibitors, targeted drugs, glucocorticoid

## Abstract

**Objective:** To summarize the situation of antineoplastic agents-induced interstitial lung diseases (ILD), provide reference for strengthening clinical management of druginduced interstitial lung diseases (DILD).

**Methods:** We retrospectively investigated the medical records of 12 patients with antineoplastic agents-induced ILD in a hospital between January and December 2020. Data collected included patients’ characteristic (gender, age, ECOG PS score, smoking history, primary tumor, concurrent diseases or complications.) and treatment conditions (DILD-causing drugs, clinical symptoms, chest CT, DILD treatment drugs, onset cycle, onset time, severity of DILD, DILD course and prognosis.).

**Results:** The median age of 12 DILD cases was 68%, 66.67% of the patients were male, lung cancer accounted for 58.33% (7/12). DILD was induced by cytotoxicity drugs, targeted drugs and immune checkpoint inhibitors (ICIs), of which ICIs accounted for 66.67% (8/12). Scattered patchy, cord-like, grid-like or flocculent shadows were observed on chest CT, mainly under the pleura of lungs. Once DILD occurs, the suspected antineoplastic agents were stopped and glucocorticoid was given, among which 83.33% (10/12) patients were treated with antibiotics. Finally, 16.67% (2/12) were cured, 33.33% (4/12) were improved, 16.67% (2/12) were not cured and 33.33% (4/12) were dead.

**Conclusion:** Antineoplastic agents-induced ILD is mostly found in elderly male lung cancer patients with smoking history. The clinical symptoms of DILD are diverse and lack of specificity. ICIs-ILD has the characteristic of high incidence and poor prognosis compared with other antineoplastic agents. Comprehensive evaluation before medication, regular review, early and adequate glucocorticoid shock therapy after onset can improve the prognosis of DILD patients.

## 1 Introduction

Interstitial lung disease (ILD) is a group of heterogeneous diseases caused by multiple etiologies, with interstitial cell proliferation, interstitial matrix hyperplasia and chronic inflammatory cell infiltration as the main pathological changes. Clinically, it is mainly manifested as progressive dyspnea, restrictive ventilation dysfunction with reduced dispersion function and hypoxemia. Imaging diffuse or multifocal distributed lesions of both lungs, and eventually develop into diffuse pulmonary fibrosis and honeycomb lung (Meyer, 2014; Conte et al., 2022). The American Thoracic Society (ATS) and the European Respiratory Society (ERS) classified ILD into four categories based on etiological, clinical, and pathological characteristics: 1) ILD of known cause; 2) Idiopathic interstitial pneumonia; 3) granulomatous ILD; 4) Other rare ILD, of which known causes of ILD include drug-related. Drug factors in the United States account for 1.9%–3.5% of all ILD (Distefano et al., 2020), while the incidence of DILD in China is underestimated. At present, hundreds of drugs have been known to cause DILD, including anti-tumor drugs, anti-microbial drugs and anti-vascular drugs, etc. In this study, we retrospectively analyzed the medication of ILD caused by anti-tumor drugs in our hospital in 2020, providing a reference for strengthening the management of ILD caused by anti-tumor drugs in clinic.

## 2 Materials and Methods

### 2.1 Data source

We used rational drug use system to extract the medical records of patients who received hormone therapy in the Department of Oncology of our hospital from January to December 2020, we consulted medical records through the HIS system, basic information of patients(including gender, age, Eastern Cooperative Oncology Group performance status (ECOG PS) score, smoking history, primary disease, concurrent diseases or complications) were collected, and the treatments of patients (including DILD-causing drugs, onset cycle, onset time, severity of DILD, clinical symptoms, chest CT, DILD treatment agents, DILD course and prognosis) were summarized.

### 2.2 Criteria for inclusion and exclusion

The diagnosis of ILD caused by antineoplastic agents is suspected in the presence of exposure to a drug known to cause lung toxicity and after exclusion of alternative causes of ILD. Diagnostic criteria are as follows: 1) recent use of antineoplastic agents; 2) clinical manifestations, imaging or pathological features suggest ILD; 3) according to the ADR correlation evaluation (National Center for ADR Monitoring, China, 2017), the time sequence of drug exposure and ILD is reasonable, the reaction stops or improves after drug withdrawal, the reaction reappears after the drug is re-administered, if there are literatures supporting that it is ADR of this drug, the evaluation is “define”; if there is a combination of drugs, but it is almost to exclude the ADR caused by those, the evaluation is “probable”; if the progressive factors of primary diseases cannot be excluded, the evaluation is “possible”; 4) antibiotic treatment is ineffective, symptoms can be relieved and shadows disappear or weaken after hormone treatment; 5) hematological test and bacterial culture were negative. Inclusion criteria: 1) meet the above three rating levels; 2) perform high- resolution computed tomography (HRCT) examination of the lungs; 3) complete clinical data.

Exclusion criteria: 1) previous radiotherapy;2) basic pulmonary diseases (such as chronic obstructive pulmonary disease, pulmonary fibrosis, ILD); 3) infection, heart failure, pulmonary embolism and other diseases leading to dyspnea; 4) alcoholism or mental illness.

### 2.3 Data processing

Descriptive statistical analysis of data using Microsoft Excel 2019.

### 2.4 Ethics statement

The studies involving human participants were reviewed and approved by the Institutional Review Board of First People’s Hospital of Lianyungang. The patients provided their written informed consent to participate in this study.

## 3 Results

### 3.1 Characteristics of patients

Through the rational drug use system, a total of 1043 patients who used glucocorticoids in the department of oncology from January to December 2020 were extracted, there were 854 cases remain after duplicate removal, through inclusion and exclusion criteria screening, 12 cases meeting the criteria were selected. The basic characteristics of patients are shown in Table 1. The median age was 68 years (range: 23–78 years), and the ratio of male to female was 2:1, 7 (58.33%) had smoking history, and 9 (75.00%) had other underlying diseases. Lung cancer accounted for more than half of the primary tumors (58.33%), followed by esophageal cancer (25.00%).

**TABLE 1:** Characteristics of patients with DILD.

Characteristics	N	%
**Sex**		
Male	8	66.67
Female	4	33.33
**Age (years)**		
<60	2	16.67
≥60	10	83.33
**ECOG PS**		
≤1	5	41.67
>1	7	58.33
**Smoking history**		
Ex/current	7	58.33
Never	5	41.67
**Primary tumor**		
Lung cancer	7	58.33
Esophageal cancer	3	25.00
B-cell lymphoma	1	8.33
Retroperitoneal leiomyosarcoma	1	8.33
**Concurrent diseases/Complications**		
Yes	9	75.00
No	3	25.00

### 3.2 Treatment of patients

**FIGURE 1:**
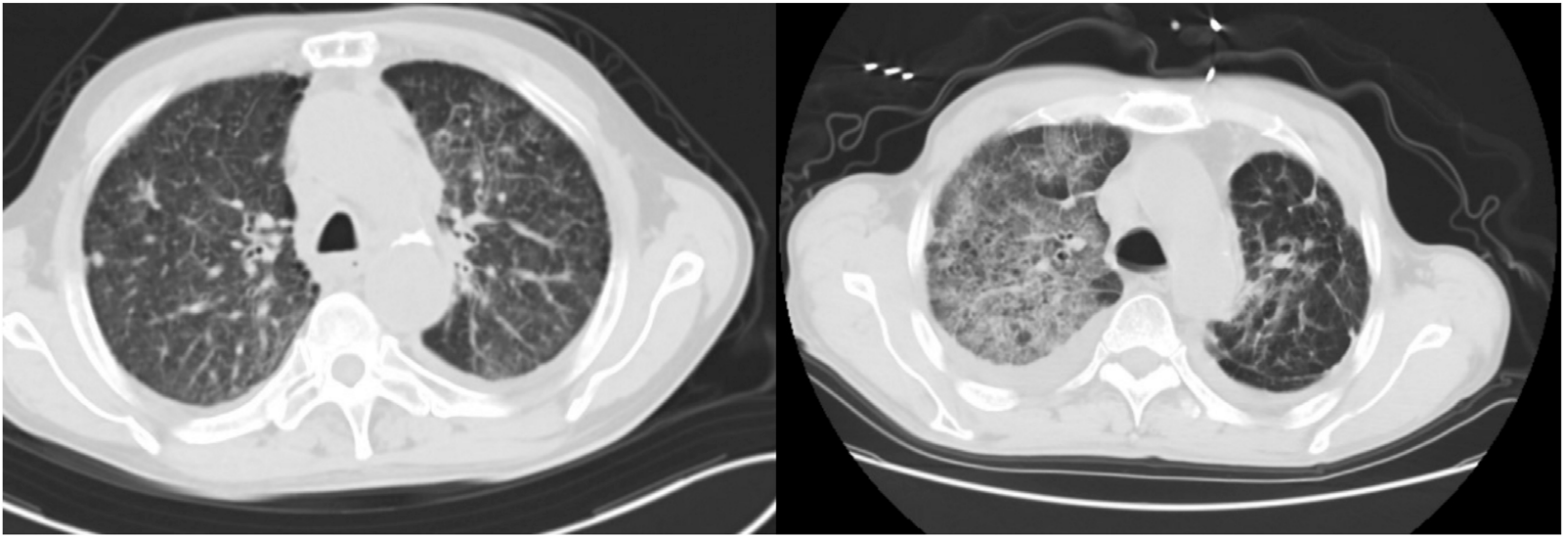
Chest HRCT of case 1 and case 2.

Treatment of DILD patients is summarized and shown in Table 2, the specific descriptions are as follows in combination with Table 2:

1. Drug suspected of inducing DILD: DILD-inducing drugs involved 8 cases of ICIs(66.67%), including Pembrolizumab Camrelizumab and Sintilimab,which were programmed death receptor-1(PD-1)inhibitors, 3 cases of targeted drugs and 1 case of cytotoxic drugs.
2. Cycle to onset: The onset cycle was the cycles of using DILD-inducing drugs, the fluctuation range was 1–8, and the median was 2.5. The ICIs-ILD most often occurred after the first cycle of treatment. The onset time of cases 2 and 3 was 4 months and 2 years after treatment respectively, who took small molecule targeted drugs orally.
3. Time to onset: The time between the initial of DILD-related symptoms and the last medication was recorded as the time to onset. The median time was 16.5 days, ranging from 1 day to 63 days.
4. The severity of DILD: The severity of DILD was determined by the Common Terminology Criteria for Adverse Events Version 5.0. All 12 patients with DILD had clinical symptoms, including 6 cases of Grade 2 (50.00 %), 4 cases of Grade 3 (33.33 %) and 2 cases of Grade 4 (16.67 %).
5. Clinical symptoms: The main clinical symptoms of DILD were cough or with a bit of sputum and wheezing, which were aggravated after activity. Cases 8 and 11 had fever as well, up to 39.2°C.
6. Imaging performance:All the 12 patients underwent chest HRCT examination, patchy, streak-like, grid-like and flocculent shadows scattered, multiple or diffusely distributed in both lungs, especially in the subpleural area. Chest HRCT of case 1 and case 2 were shown in Figure 1.
7. Treatments: All DILD patients were treated with glucocorticoid, while symptomatic treatments such as oxygen inhalation, relieving cough, relieving asthma and eliminating phlegm, and correcting acid-base balance were given at the same time. Different types of glucocorticoids are involved in the treatment, all hormone doses were converted to methylprednisolone equivalent doses according to the ratio of methylprednisolone : hydrocortisone : hydrocortisone =4:5:20. Finally, the dose of methylprednisolone was ranging from 40 mg∙d^−1^ to 160 mg∙d^−1^, with a median dose of 80 mg·d^−1^. 10 patients were given antibiotics concurrently, mainly β-lactamase inhibitor compound (7/10).
8. Course of DILD:The course of DILD was the duration of hormone therapy,with a fluctuation range of 10–103 days, and the median was 35.5 days.
9. Prognosis:After treatment, 16.67% (2/12) were cured, 33.33% (4/12) were improved, 16.67% (2/12) were not cured and 33.33% (4/12) were dead among patients with DILD. Due to long-term application of high-dose glucocorticoid,case 4 had secondary diabetes, osteoporosis and fungal infection.

**TABLE 2:** Treatment of patients with DILD.

NO.	Drug suspected of inducing DILD		Onset cycle	Onset time (d)	ADR grade	Clinical symptoms	HRCT	Treatments		DILD course (d)	Prognosis
								Antibiotics	Glucocorticoid (MP dose mg∙d^-1^)		
1	Cytotoxic drugs	nab-PTX	2	33	G2	cough with sputum	diffuse grid and patchy shadows in both lungs	piperacillin- tazobactam	80	10	not cured
2	Targeted drugs	Osimertinib	2 years	/	G3	cough and wheezing, worsening after activity	flake and patchy shadows in both lungs	moxifloxacin+ linezolid	80	103	dead
3	Targeted drugs	Crizotinib	4 months	/	G2	cough with a little sputum	multiple flocculent blurs in both lungs	piperacillin-sulbactam	40	16	improved
4	Targeted drugs	Rituximab	2	7	G3	cough and wheezing	multiple patchy shadows in subpleural lung	biapenem	160	69	dead
5	ICIs	Pembrolizumab	1	1	G4	worsening wheezing with chest tightness	scattered flocculent shadows in both lungs	cefoperazone-sulbactam	160	26	dead
6	ICIs	Pembrolizumab	1	1	G2	wheezing	small patchy shadows in both lungs	——	40	36	cured
7	ICIs	Pembrolizumab	8	17	G4	cough with sputum, worsening after activity	multiple patchy density increase in both lungs	cefotaxime-sulbactam	160	64	dead
8	ICIs	Camrelizumab	3	63	G3	worsening cough with wheezing and low-grade fever	patchy blurred shadows in both lungs	cefotaxime-sulbactam	120	47	improved
9	ICIs	Camrelizumab	1	16	G2	cough with sputum	patchy shadows in right lung	cefotaxime-sulbactam	40	10	improved
10	ICIs	Camrelizumab	4	18	G3	cough with sputum	grid and strip shadows in subpleural lung	piperacillin-sulbactam	80	27	not cured
11	ICIs	Sintilimab	1	4	G2	cough and wheezing with high fever	spotted blurred shadows in both lungs	biapenem + vancomycin	120	50	cured
12	ICIs	Sintilimab	4	38	G2	cough with sputum	grid shadows in both lower lungs	——	40	35	improved

ICIs: Immune checkpoint inhibitors, nab-PTX: albumin-bound paclitaxel, MP: Methylprednisolone, /means not applicable, ——means no antibiotics used.

## 4 Discussion

### 4.1 Mechanism of drug-induced interstitial lung diseases induced by antineoplastic agents

Skeoch et al. (2018) reviewed 1694 literatures about ILD and found that antineoplastic agents accounted for 23–51% of all DILD,with the characteristic of low incidence and high mortality. The DILD-induced drugs in this research involved a total of 7, and all the drug instructions indicated the possibility of ILD. The pathogenesis of DILD is poorly understood, but several mechan isms show that ILD induced by cytotoxic drugs may be a direct damage to alveolar epithelial or capillary endothelium cells, or an indirect damage caused by the recruitment of cytokines or inflammatory factor (Ryrfeldt, 2000). Alveolar type II epithelial cells express epidermal growth factor receptor (EGFR), which take part in the repair of alveolar wall. Targeted drugs inhibit not only the growth of tumor, but also the growth of tracheal epithelial cells and the repair of injury, which aggravates lung damage. In addition, targeted drugs may cause damage of alveolar and bronchial epithelial, leading to chronic inflammation, both of which stimulate fibroblast migration, proliferation, and production of extracellular matrix, thereby causing pulmonary fibrosis (Matsuno, 2012). Rituximab-ILD may be caused by the fact that it binds to and kills B cells, resulting in the release of TNF-α IFN-α IL-6 IL-8 and other cytokines by T lymphocytes (Lands, 2010), it may also be caused by a type III hypersensitivity reaction triggered due to its immunogenicity (Lioté et al., 2010). The development of ICIs-ILD may involve dysregulation of immune effector molecules and T cells in the pulmonary interstitium, leading to subsequent inflammatory responses (Delaunay et al., 2017).

### 4.2 Risk factors for drug-induced interstitial lung diseases

The occurrence and development of ILD induced by antineoplastic agents is still unpredictable, which may be related to many factors, including drug factors and non- drug factors. Drug factors: ^①^type of drugs; it has been reported that patients receiving targeted and ICIs therapy have a higher incidence of DILD and a worse prognosis than cytotoxic drugs (Nishino et al., 2017), and the incidence of ILD caused by PD-1 inhibitors is higher than that caused by programmed death ligand-1 (PD-L1) inhibitor. ^②^drug interaction; concomitant use of two or more drugs with pulmonary toxicity is associated with an increased risk of DILD. Non-drug factors: ^①^Age; the elderly are more likely to have serious adverse reactions due to reduced renal excretion function and reduced liver blood flow, as well as changes in overall metabolic function. ^②^Sex; Kaku et al. (2022) found that male sex was associated with a higher risk of severe DILD-related death. ^③^Smoking history; the risk of DILD of patients who smoked previously is higher than those not (Ando et al., 2006). ^④^Lung underlying diseases; it has been reported that approximately 20%–24% of patients with preexisting ILD will develop DILD (Isobe et al., 2010). ^⑤^Radiotherapy; radiotherapy destroys DNA damage and repair proteins involved in the repair of lung injury, especially when combined with chemoradiation, which greatly increases the risk of lung toxicity. ^⑥^Type of cancers; many lung cancer patients have coexisting ILD, and these patients have a high risk of developing DILD (Raghu et al., 2004), squamous cell carcinoma was also identified as a significant risk factor in the univariate analysis (Sakurada et al., 2015). Among the 12 patients with DILD in this research, patients over 60 years old accounted for 83.33%, 7 patients with lung cancer, the ratio of male to female was 2:1, and the most of the male patients had a history of smoking, all of 8 patients with ICIs- ILD were induced by PD-1 inhibitors, which was consistent with results of studies on risk factors for DILD. The case 11 had a history of psoriasis, and ILD occurred after the first cycle of immunotherapy. Therefore, it is essentital to choose drugs with low pulmonary toxicity in patients with advanced age and smoking history, ICIs is especially prudent for patients with autoimmune diseases.

### 4.3 Onset time and clinical symptoms of drug-induced interstitial lung diseases

The time to onset of ILD after initiation of antineoplastic agents ranges from a few days to several years, and the pulmonary toxicities often present with relatively non- specific features. Symptoms might include dyspnoea, hypoxia, cough, chest discomfort, or, less commonly, fever, a few patients with DILD have no clinical symptoms just imaging abnormalities (Rashdan et al., 2018), ground-glass nodules or patchy nodular infiltration in the lower lobes of both lungs was detected in the CT findings of these patients. It was reported that the median onset time of ICIs-ILD is 2.8 months (Naidoo et al., 2017), ILD induced by Osimertinib occurs after 3 months while nab-PTX- induced ILD occurs from weeks to months after treatment (Tan et al., 2020). In summary, ILD occurs later than other adverse drug reactions, while combination therapy occurs earlier. The clinical manifestations of the 12 patients with DILD in this research were different, including cough (83.33%), sputum cough (50.00%), wheezing (58.30%), chest tightness (8.33%) and fever (16.67%), while HRCT findings of new patchy or cord-like blurred shadows compared with before. The earliest onset time of DILD was one day after the first cycle of Pembrolizumab, and the latest was 2 years after the medication of Osimertinib. The onset time of ILD induced by the same drug in disparate patients is also different. Therefore, when patients use drugs with pulmonary toxicity, it is necessary to pay attention to new discomfort and perform chest CT routinely, so as to achieve early detection and early treatment, and reduce the adverse effects of DILD on patients.

### 4.4 Treatment and prognosis of drug-induced interstitial lung diseases

The goal of ILD treatment is to suppress the inflammatory response, promote exudation absorption, prevent pulmonary interstitial fibrosis, and protect cardiopulmonary function. Refer to the diagnosis and treatment guidelines and expert consensus of ILD induced by antineoplastic drugs at home and abroad (Zhang et al., 2021; Chinese Society of Clinical Oncology, 2022; National Comprehensive Cancer Network, 2023), the treatment strategies are formulated as follows: 1) Discontinuation of (and avoidance of further exposure to) the culprit drug is themainstay of treatment; 2) Patients with confirmed or suspected to have DILD are generally treated with glucocorticoid (methylprednisolone 0.5–4.0 mg/(kg∙d) or equivalent according to severity grade), and plan a slow glucocorticoid taper over ≥ 6 weeks. It is also important to supplement calcium and vitamin D, monitor levels of oxygen saturation, blood pressure, blood sugar, and prevent gastrointestinal bleeding; 3) Anti-infective therapy is recommended if co-infection cannot be ruled out (sensitive anti-infective drugs are selected on demand or based on microbiological findings); 4) Oxygen therapy: It is recommended that ILD patients with resting hypoxemia receive long-term oxygen therapy for more than 15 h/d according to the indications for oxygen therapy in chronic obstructive pulmonary disease. For ICIs- ILD patients with Grade3-4,if no improvement is observed after 48 hours of treatment, consider additional immunosuppression with any of the following agents: infliximab, IVIG, or mycophenolate mofetil. 12 patients with DILD in this research discontinued suspect drugs and started glucocorticoid immediately, 10 of them in combination with antibiotics. The patients who were treated with adequate hormone according to the principle of treatment accounted for 66.67% (8/12), among which 50% (4/8) died, while the patients with low- dose hormone improved or cured eventually. This means that the prognosis of patients with DILD in this research is not directly related to the dose of hormone, but may be related to the severity of DILD and the sensitivity of the body to glucocorticoid. The prognosis of patients with severe disease or low response to hormone is poor. Two of the four death cases in this research were caused by Pembrolizumab, ICIs were used as second- or third-line treatment while cytotoxic agents were first-line in lung cancer, it may be a reason for the worse prognosis of ICIs-ILD than ILD induced by cytotoxic agents.

### 4.5 Rechallenge with causative drugs

All patients with DILD in this research discontinued suspected drugs permanently. Up to now, there is no international consensus on the risks and benefits of rechallenge after ILD induced by antineoplastic agents. Refer to NCCN guideline Version1. 2023- management of immunotherapy-related toxicities (National Comprehensive Cancer Network, 2023) and FUSCC criteria for the management of targeted drug-induced interstitial lung disease in solid tumors (Zhang et al., 2021),for ILD induced by targeted drugs, Grade4 is recommended to be discontinued permanently, while Grade2-3 is recommended to be reduced by one therapeutic dose level after recovery; it is recommended to discontinue ICIs for life for Grade3-4 ICIs-ILD, and ICIs can be rechallenged after recovery from Grade2 ICIs-ILD. Gu et al. (2021) had reported a case of successful Osimertinib rechallenge after recovery from Osimertinib-induced ILD in a patient with EGFR-mutant non-small cell lung cancer, which experienced Grade2 ILD and recovered after glucocorticoid therapy for 13 days. The patient received Osimertinib treatment (80 mg qd) again, and oral prednisone was given concurrently, there was no disease progression or ILD recurrence within more than 16 months of treatment. Clinically, if the drug is essential and could not be replaced, rechallenge should always be discussed with a multi-disciplinary team.

## 5 Conclusion

DILD is a rare adverse reaction of antineoplastic drugs. Due to the lack of specificity of clinical manifestations and different onset time, patients often miss the best treatment time and affect the survival time. Accurate identification of risk factors can help screen high-risk patients before treatment with relevant drugs. During the treatment period, it is necessary to closely monitor the changes of the disease condition, especially the respiratory function and chest CT. Meanwhile, the medication education for the patients should be strengthened to improve the cognitive level of the patients. Once the disease condition changes, early identification, early diagnosis and timely treatment can avoid serious consequences. It is equally important to prevent the complications such as osteoporosis, gastrointestinal bleeding, and fungal infection during the treatment of DILD. However, the decision on whether to rechallenge the same causative drug after remission of DILD requires careful consideration of risks and benefits as well as the availability of alternative treatments.

## Data Availability

The original contributions presented in the study are included in the article/Supplementary material, further inquiries can be directed to the corresponding author.
